# Seroprevalence and determinants of transfusion transmissible infections among voluntary blood donors in Homabay, Kisumu and Siaya counties in western Kenya

**DOI:** 10.1186/s13104-018-3276-y

**Published:** 2018-03-12

**Authors:** Calleb George Onyango, Lilian Ogonda, Bernard Guyah, Peter Okoth, Clement Shiluli, Felix Humwa, Vallarie Opollo

**Affiliations:** 1 Department of Biomedical Science and Technology, Maseno University, Maseno, Kenya; 2 Jaramogi Oginga Odinga Teaching and Referral Hospital, Kisumu, Kenya; 3 Center for Disease Control and Prevention, Kisumu, Kenya; 4 Regional Blood Transfusion Center, Kisumu, Kenya; 5 Kisumu-Kakamega Road, P.O Box 849, Kisumu, Code 40100 Kenya

**Keywords:** Seroprevalence, HIV, HBV, Donors, Kenya

## Abstract

**Objective:**

Since the implementation of a series of blood donation safety improvements in Kenya, information about seroprevalence and determinants of transfusion transmissible infections among voluntary blood donors especially in high HIV burden regions of Homabay, Kisumu and Siaya counties remain scanty. A cross-sectional study examining HIV, syphilis, hepatitis B and C virus sero-markers and associated determinants was conducted among voluntary blood donors. Their demographic characteristics and previous risk exposure were recorded in a pre-donation questionnaire, while blood samples collected were screened for hepatitis B, hepatitis C, human immunodeficiency viruses by ELISA and RPR (syphilis), then confirmed using CMIA.

**Results:**

Overall TTIs seroprevalence was 114 (9.4%), distributed among HIV, HBV, HCV and syphilis at 14 (1.15%), 42 (3.46%), 39 (3.21%) and 19 (1.56%), respectively, with co-infections of 3 (0.25%). There were no significant differences in proportions distributions among demographic variables. However, high risk sex was significantly associated with higher odds of HBV infections [> 1 partner vs. 0–1 partner; odd ratio (OR) 2.60; 95% confidence interval (CI) 1.098–6.86; p = 0.046]. In conclusion, a substantial percentage of blood donors still harbor transfusion transmissible infections despite recent safety improvements with greater majority cases caused by HBV infections arising from previous exposure to high risk sex.

**Electronic supplementary material:**

The online version of this article (10.1186/s13104-018-3276-y) contains supplementary material, which is available to authorized users.

## Introduction

Transfusion transmissible infections especially human immunodeficiency virus and hepatitis viruses, are a constant threat to blood safety for the recipient, and more endemic in Africa, thus making donors in this region vulnerable to risk of exposure [1]. Moreover, co-infection is also common due to similar routes of transmission [2], with prevalence varying with time and regions [3]. Infections with HIV compromises immunological status of a person, hepatitis B and C viral infections cause liver cirrhosis and hepatocellular carcinoma while infections with syphilis results in neonatal mortality [4]; thus, high quality screening for the four major TTIs have become mandatory and irreducible minimum that must be tested in all blood donations to achieve safety threshold [5].

Globally, about 1.6 million blood units are destroyed annually owing to TTIs seropositivity [5], with 10% discarded in sub-Saharan Africa [6]. In the neighbouring Democratic Republic of Congo (DRC), risk of transfusion-transmitted HIV, HBV and HCV was estimated at 0.6, 7.9 and 3.1 infections per 1000 donations, respectively [7]. Here in Kenya, an estimated risk of transfusion-transmitted HIV was reported at 2% [8]. Moreover, despite of recent blood donation safety improvements such as stringent pre-donation screening, enhanced haemovigilance through e-progesa blood computerized system (BECs) and implementation of fourth generation p24 antigen and HIV antibody screening assays [9], a substantial amount of blood units estimated at 5.26%, are still discarded annually owing to TTIs seropositivity [10]. Furthermore, HIV, HBV, HCV and syphilis seropositive blood discards estimated at 2.6, 3.9, 2.2 and 0.5%, respectively, were reported earlier in Kisumu region under Kenya national blood transfusion services (KNBTS) covering Homabay, Kisumu and Siaya counties in western Kenya [11]. The three counties experience the greatest burden of HIV and other co-infections in the region [12], but also serve as a major blood basket supplying most hospital in the region based on their proximity to regional blood donor center of Kisumu and ease for transport during donations. To date, there is no published data on blood donation safety assessment following recent safety improvements.

Socio-demographic characteristics influence the distribution of TTIs among blood donors. In Kenya, national blood transfusion services (NBTS) rely mainly on young voluntary blood donors, particularly secondary schools, colleges and University students, majority in the age range of 15–24 years [13]. Blood donations from school students are preferred over adult donors owing to lower HIV prevalence estimated at 1% compared to 6.6% prevalence recorded in adults aged 30–34 years [13]. However, a study carried out in Kenya using stimmunology still detected a significant number of early pre-seroconversion of HIV carriers both among adults and teenage population [14]. This study sought to establish the dominant demographic and other risk factors still playing a role in TTIs seropositivity recorded in the region despite recent safety interventions.

## Main text

### Methods

Using Yamane formula, and based on KNBTS selection protocol, blood samples of 1215 voluntary blood donors aged 16–65 years from Homabay, Kisumu and Siaya counties (see Additional file 1), was determined for a cross-sectional study by random sampling. Laboratory tests were conducted at Kisumu Regional Blood Donor Center, Kenya, from November 2015 to October 2016 based on its proximity to study sites and quality standards available. Ethical approval was obtained from Maseno University Ethical Review Committee (MUERC). Blood donors were enrolled under informed written consent or with guardian consent in case of donors less than 18 years. A self administered pre-donation questionnaire (see Additional file 2), was used to screen potential donors for health fitness and to collect demographic details such as age. Recent exposure to high risks such as unprotected sex in a period less than 3 months were noted and excluded. Baseline hemoglobin test and body weight measurements was performed by nurse counselor to exclude individuals with anemia (hemoglobin < 12.5 g/dl) or body weight below 50 kg. After blood collection, about 10 ml of donor blood was transferred into a plain vacutainer tube (BD Franklin Lakes, USA) for HIV-1/2, HBV, HCV and syphilis serological testing.

Sera were tested for antigen or/antibody to HIV-1/2 using Vironostika HIV Uni-Form II Ag/Ab test kit (Biomerieux SA, Microelisa system), antibodies to hepatitis C (Anti-HCV) using Abbott Murex Anti-HCV version 4.0 (Murex Biotech SA (Pty) Ltd) and Hepatitis B surface antigen (HBsAg) using Hepanostika HBsAg Ultra test (Biomerieux SA, Microelisa system). Sera were also tested for presence of treponemal antibodies using rapid plasma reagin (RPR) test (Omega diagnostics, UK). All tests were done according to manufacturers’ instructions. All reactive samples were confirmed by chemiluminescent immunoassay. A result was considered positive if the first test and confirmatory test were all seroreactive.

Statistical analysis was performed using SPSS version 20. Seroprevalence distribution in subcategories were compared using Chi square test while logistic regression was used to determine the association between TTIs seroprevalence and various risk factors with a p-value < 0.05 considered statistically significant.

### Results

The baseline characteristics of the study participants are summarized in Table 1. Of 1215 voluntary blood donors recruited, 700 (57.6%) were males and 515 (42.4%) females. The overall seroprevalence of TTIs was 114 (9.4%), distributed among HIV, HBV, HCV and syphilis variables at 14 (1.15%), 42 (3.46%), 39 (3.21%) and 19 (1.56%), respectively, with co-infections detected in only 3 (0.25%). There were no significant differences in proportions distributions among demographic variables tested. Demographic and risk factors among donors were evaluated as shown in Table 2. High risk sex was significantly associated with higher odds of HBV infections [> 1 partner vs. 0–1 partner; odd ratio (OR) 2.60; 95% confidence interval (CI) 1.098–6.86; p = 0.046], rather than other infections (see Additional file 3). All other characteristic variables tested had no significant association with infections.

**Table 1:** Demographic characteristics of reactive and non-reactive participants

Characteristics	Non-reactive, n (%)	Reactive, n (%)
Age groups		
Teenage (16–19)	894 (91.6%)	82 (8.4%)
Adults (20–65) years	211 (88.28%)	28 (11.72%)
Gender		
Females	475 (92.23%)	40 (7.77%)
Males	630 (90.0%)	70 (10.0%)
Marital status		
Married	49 (98.0%)	1 (2.0%)
Single	1056 (90.64%)	109 (9.36%)
Number of donations		
First	813 (90.33%)	87 (9.76%)
Repeats	292 (92.7%)	23 (7.30%)
Counties		
Homabay	293 (89.0%)	36 (10.94%)
Kisumu	469 (92.69%)	37 (7.31%)
Siaya	343 (90.26%)	37 (9.74%)

Data shown are numbers (n) and proportions (%) of TTIs seronegative (1105) and seropositive (110) as distributed among demographics

**Table 2:** Demographic variables of individual infections among reactive and non-reactive blood donors

Characteristics	HIV (n = 14)		p-value	HBV (n = 42)		p-value	HCV (n = 39)		p-value	Syphilis (n = 19)		p-value
	Non-reactive	Reactive		Non-reactive	Reactive		Non-reactive	Reactive		Non-reactive	Reactive	
Counties												
Homabay	322	7	0.062	317	12	0.824	318	11	0.633	323	6	0.192
Kisumu	503	3	ref.	489	17	ref.	492	14	ref.	502	4	ref.
Siaya	376	4	0.45	367	13	0.96	366	14	0.441	371	9	0.66
Age groups years												
Teenagers (16–19)	965	11	ref.	945	31	ref.	947	29	ref.	961	15	ref.
Adults (20–65)	236	3	0.863	228	11	0.282	229	10	0.348	235	4	0.879
Gender												
Female	510	5	ref.	501	14	ref.	498	17	ref.	508	7	ref.
Male	691	9	0.613	672	28	0.23	678	22	0.877	688	12	0.623
Marital status												
Married	50	0		49	1	ref.	50	0		50	0	
Single	1151	14	–	1124	41	0.57	1126	39	–	1146	19	–
Drug use												
No	1189	14		1161	42		1164	39		1185	18	ref.
Yes	12	0	–	12	0	–	12	0	–	11	1	0.095
High risk sex												
No	1139	13	ref.	1115	37	ref.	1116	36	ref.	1134	18	ref.
Yes	62	1	0.741	58	5	*0.046*	60	3	0.476	62	1	0.988
No. donations												
First	888	12	ref.	869	32	ref.	869	31	ref.	884	16	ref.
Repeats	313	2	0.329	305	10	0.75	307	8	0.435	312	3	0.317
Blood transfusion												
No	1180	14		1153	41	ref.	1157	37	ref.	1175	19	
Yes	21	0	–	20	1	0.742	19	2	0.118	21	0	–

High risk sex was significantly associated with HBV infections p = 0.046

Data shown are: ref., reference category; n, numbers; HIV-1/2Ag/Ab, human immunodeficiency virus 1 and 2 antigen and antibody; HBVsAg, hepatitis B surface antigen; Anti-HCV, antibody to hepatitis C virus; –, no analysis done due to lack of cases; No, not exposed; Yes, exposed; Reactive, seropositive; Non-reactive, seronegative

### Discussion

The present study was set to determine the seroprevalence and determinants of HIV, HBV, HCV and syphilis among voluntary blood donors in Homabay, Kisumu and Siaya counties in western Kenya. Results established that, overall seroprevalence of TTIs among voluntary blood donors was 9.4%. Similar results have been reported in studies done in Ethiopia showing 9.5% seroprevalence [1], and in Benin, Nigeria showing 12.5% seroprevalence [15]. However, different result were reported in some parts of Nigeria showing seroprevalence of 19.0% [16], and in Burkina Faso showing seroprevalence of 24% [17]. This observation relates to HIV endemicity and intermediate endemicity of HBV and HCV in the region. In this study, overall TTIs seroprevalence in the three study sites was relatively higher compared to earlier reports of 5.26% national seroprevalence [10]. This variation may be attributed to HIV endemicity experienced in the three study sites as compared to the rest of the country showing difference in HIV/STIs profile. Meanwhile, co-infection of 0.25% detected in the study population was comparable to 0.02% reported earlier in a local study [18], and 0.8% reported in the neighbouring Ethiopia [1].

**Fig. 1:**
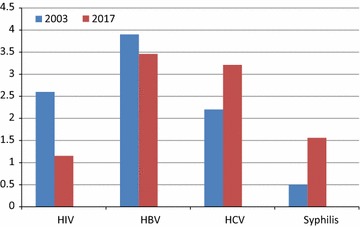
TTIs seroprevalence of 2003 [11] was compared with current study 2017 using test of proportions. HIV infections decreased significantly (p < 0.001), whereas HBV, HCV and syphilis remained similar

The HIV seroprevalence of 1.15% detected among blood donors is indicative of low seroprevalence and decreased susceptibility to HIV infections. Similar studies in DRC reported similar result of 1.1% seroprevalence [7]. However, in Ethiopia, a relatively higher seroprevalence of 3.8% was reported [1]. These findings were comparable to the previous national estimates of 0.96–1.43% seroprevalence reported for the periods 2007–2010 [19], and 0.5–1.5% seroprevalence for the periods 2011–2012 [20]. Likewise, the result was also comparable to other national estimates of 1.2–2.5% seroprevalence reported earlier in a local study [21]. This study observation could be results of exclusion of family replacement donors (FRDs) from the study, and the milestones of beyond zero campaign initiatives in the country. In contrast, the findings were relatively low compared to 2.6% national seroprevalence reported in a similar local study [13], as well as 2.6% seroprevalence observed earlier from same study sites [11], (Fig. 1).

In this study, HBV seroprevalence of 3.46% detected among blood donors is indicative of intermediate endemicity and increased susceptibility to HBV infections. Similar results have been reported in Ethiopia showing seroprevalence of 4.7% [1], and in Egypt showing seroprevalence of 4.3% [22]. The findings were comparable to 3.9% seroprevalence reported earlier from the same study sites [11], but relatively higher compared to 1.97–2.77% national estimates for the periods 2006–2010 [19]. In contrast, these study findings were relatively lower compared to the national seroprevalence peak of 5.2% reported in the period 2011–2012 [20]. Thus, it is evident from the study that, despite of recent blood donation safety improvements, HBV infection remains a safety concern in blood donation.

The HCV seroprevalence of 3.21% detected among voluntary blood donors is indicative of intermediate endemicity and increased susceptibility to HCV infections. Similar studies reported relatively lower seroprevalence of 2.7% in Egypt [22], and 1.8% in Cameroon [23] compared to this study results. The variation observed in HCV seroprevalence may be attributed to difference in geographical settings and study methodology adopted by different authors. In this study, HCV seroprevalence was relatively higher compared 0.79–0.99% national estimates for the period 2007–2010 [19]. In addition, this study result was relatively higher compared 2.2% seroprevalence previously reported in the same study sites [11]. Thus; it is evident from the study that HCV infection remains a challenge in blood donation safety.

Syphilis seroprevalence of 1.56% detected among blood donors is indicative of low seroprevalence and decreased susceptibility to syphilis infection. Similar studies in Ethiopia reported similar results of 1.3% seroprevalence [1]. However, different results were reported in Ghana showing seroprevalence of 13.5% [24], and in Cameroon showing 9.1% seroprevalence [25]. Variation observed in seroprevalence may be attributed to difference in geographical setting. In this study, syphilis seroprevalence was similar to the national estimates of 0.15–0.28% [19], and 0.5% seroprevalence reported earlier in the same study area [11]. This observation may be attributed to milestones of beyond zero campaign initiatives.

In the analysis of demographic and risk factors, HBV infection was significantly associated with previous exposure to high risk sex. This may be explained by the low economic status initiating young adolescents to multiple sexual relationships, thus making them vulnerable to HBV and other co-infections. The findings corroborate local studies that found sex for cash payment associated with HIV and other co-infections [12]. In contrast, different results were observed in Egypt showing that TTIs was not associated with high risk sex [26]. In relation to age and gender, none was associated with any of the TTIs seroprevalence, although previous local studies had reported different results showing that adults’ aged ≥ 20 years were more likely to get infected by HIV compared to young adolescents aged 10–19 years [12]. Moreover, females were more likely to test HIV positive compared to males [13]. The variation observed may be related to the difference in study population. In relation to blood transfusion history, there was no association with any of the TTIs. However, a similar study in Egypt reported different results showing HCV seroprevalence of 72% among donors with transfusion history [26]. Meanwhile, a previous local study had reported HIV transfusion transmission risk of 2% among recipients [8]. On the contrary, this study did not find any association between TTIs seroprevalence and blood transfusion history. Recent implementation of high quality screening assays, coupled with stringent pre-donation screening, and enhanced haemovigilance may have influenced decrease in transfusion risk observed earlier. Meanwhile, previous exposure to illicit drug use was not associated with TTIs seroprevalence. However, different results have been reported in Egypt showing 47.5% of blood donors with drug use history testing HCV positive [26].

In conclusion, a substantial percentage of blood donors still harbor transfusion transmissible infections despite recent blood donation safety improvements with greater majority cases caused by HBV infection arising from previous exposure to high risk sex. Promoting safe sex education in learning institutions and early uptake of HBV self testing with subsequent vaccination would help reduce TTIs burden observed among blood donors.

## Limitation

Detection of HBV was based on HBVsAg marker without considering IgM and IgG antibodies to the core protein ideal for a complete diagnosis of infection stages [27]; hence low seropositive estimate was possible. Additionally, questions on illicit drug use and sex life style are all associated with stigma with a possibility of influence on overall risk estimate.

## Additional files

**Additional file 1.** Participants’ distribution.

**Additional file 2.** Pre-donation questionnaire.

**Additional file 3.** Excel dataset for regression analysis.

## Abbreviations

AIDS: acquired immunodeficiency syndrome
CMIA: chemiluminescent micro particle immunoassay
CDC: Center for Disease Control and Prevention
ELISA: enzyme linked immunosorbent assay
FRD: family replacement donors
HBV: hepatitis B virus
HCV: hepatitis C virus
HIVAg/Ab: human immunodeficiency virus antigen/antibody
JOOTRH: Jaramogi Oginga Odinga Teaching and Referral Hospital
KNBTS: Kenya national blood transfusions services
NACC: National AIDS Control Council
RPR: rapid plasma regain
RBTC: Regional Blood Transfusion Center
TTIs: transfusion transmissible infections
WHO: World Health Organization
NASCOP: National AIDS/STI Control Program
BECs: blood establishment computerized system
