# Correction to: Seroprevalence and determinants of transfusion transmissible infections among voluntary blood donors in Homabay, Kisumu and Siaya counties in western Kenya

**DOI:** 10.1186/s13104-018-3521-4

**Published:** 2018-06-26

**Authors:** Calleb George Onyango, Lilian Ogonda, Bernard Guyah, Peter Okoth, Clement Shiluli, Felix Humwa, Vallarie Opollo

**Affiliations:** 1 Department of Biomedical Science and Technology, Maseno University, Maseno, Kenya; 2 Jaramogi Oginga Odinga Teaching and Referral Hospital, Kisumu, Kenya; 3 Kenya Medical Research Institute (KEMRI), Kisumu, Kenya; 4 Regional Blood Transfusion Center, Kisumu, Kenya; 5 Kisumu-Kakamega Road, P.O Box 849, Kisumu, Code 40100 Kenya

## Correction to: BMC Res Notes (2018) 11:171 10.1186/s13104-018-3276-y

Following publication of the original article [1], the authors reported that for two of the authors, Felix Humwa and Vallarie Opollo, an incorrect affiliation has been given. In this Correction the incorrect and correct affiliation are listed.

Originally the affiliation for Felix Humwa and Vallarie Opollo has been published as:

- Center for Disease Control and Prevention, Kisumu, Kenya

The correct affiliation is:

- Kenya Medical Research Institute (KEMRI), Kisumu, Kenya

The corrected list of affiliations is included in this Correction.

## References

[1] Onyango CG, Ogonda L, Guyah B, Okoth P, Shiluli C, Humwa F, Opollo V (2018). Seroprevalence and determinants of transfusion transmissible infections among voluntary blood donors in Homabay, Kisumu and Siaya counties in western Kenya. BMC Res Notes.

