# Supplementary material for: Seroprevalence and determinants of transfusion transmissible infections among voluntary blood donors in Homabay, Kisumu and Siaya counties in western Kenya
Source: BMC Res Notes. 2018 Mar 12;11:171. doi: 10.1186/s13104-018-3276-y (PMC5848540; doi:10.1186/s13104-018-3276-y)
Supplement: Supplementary file 1 — Additional file 1. Participants’ distribution. [file 13104_2018_3276_MOESM1_ESM.docx]

**DISTRIBUTION OF STUDY PARTICIPANTS BY COUNTY AND INSTITUTIONS**

| Counties | Institutions | Institutions  population | Number of  Donors  registered | Number of  Study  participants | Percentage  participants |
| --- | --- | --- | --- | --- | --- |
| HOMABAY | ST. INNOCENT GIRLS | 430 | 45 | 31 | 7.2% |
|  | MAWEGO GIRLS | 380 | 48 | 35 | 9.2% |
|  | LIGISA MIXED | 460 | 51 | 46 | 10% |
|  | AGORO MIXED | 350 | 43 | 39 | 11.1% |
|  | NYANGIELA MIXED | 300 | 35 | 30 | 10% |
|  | LALA MIXED | 450 | 46 | 28 | 6.2% |
|  | KOBALA MIXED | 300 | 40 | 11 | 3.7% |
|  | OTHORO MIXED | 480 | 37 | 33 | 6.9% |
|  | LUORA MIXED | 450 | 30 | 28 | 6.2% |
|  | ORERO BOYS | 900 | 86 | 13 | 1.4% |
|  |  |  |  |  |  |
|  | **COLLEGES** |  |  |  |  |
|  | MTC -KENDU ADVENT. | 180 | 51 | 15 | 8.3% |
|  | **CAMPS** |  |  |  |  |
|  | MAWEGO SDA | 200 | 20 | 20 | 10% |
|  |  |  | **TOTAL** | **329** |  |
| KISUMU | **SCHOOLS** |  |  |  |  |
|  | RIDORE MIXED | 430 | 42 | 29 | 6.7% |
|  | ALAWA MIXED | 390 | 38 | 33 | 8.5% |
|  | ST. RITA MIXED | 600 | 72 | 54 | 9.0% |
|  | DR. ALOO MIXED | 530 | 38 | 19 | 3.6% |
|  | NYAKACH GIRLS | 1500 | 76 | 53 | 3.6% |
|  | KISUMU GIRLS | 1200 | 183 | 79 | 6.6% |
|  | ST. PETER KONIN | 300 | 31 | 20 | 6.7% |
|  | WITHUR MIXED | 530 | 63 | 56 | 10.6% |
|  | ACHEGO GIRLS | 400 | 43 | 28 | 7.0% |
|  |  |  |  |  |  |
|  | **COLLEGES** |  |  |  |  |
|  | KMTC-KISUMU | 1200 | 64 | 26 | 2.2% |
|  | MASENO UNIV. | 10,000 | 225 | 27 | 0.27 |
|  | NYABONDO MTC | 200 | 34 | 11 | 5.5% |
|  |  |  |  |  |  |
|  | **CAMPS** |  |  |  |  |
|  | SPORTSGROUND | 1000 | 45 | 26 | 2.6% |
|  | PEFA MIGOSI | 600 | 62 | 10 | 1.7% |
|  | PIPELINE KENYA | 100 | 21 | 5 | 5% |
|  | JCC CHURCH | 700 | 36 | 11 | 1.6% |
|  |  |  | **TOTAL** | **506** |  |

**DISTRIBUTION OF STUDY PARTICIPANTS BY COUNTY AND INSTITUTIONS**

| County | School | School  population | Donors  registered | Study  participants | Percentage  participants |
| --- | --- | --- | --- | --- | --- |
| SIAYA | NYAMIRA GIRL | 1137 | 72 | 45 | 4.0% |
|  | MAJANGO MIX | 350 | 68 | 43 | 12.3% |
|  | SAWAGONGO | 800 | 77 | 8 | 1.0% |
|  | NYAMBARE | 360 | 43 | 24 | 6.7% |
|  | UGENYA HIGH | 500 | 47 | 19 | 3.8% |
|  | BAR-KOWINO | 550 | 56 | 24 | 4.4% |
|  | MITIRO MIX | 440 | 64 | 33 | 7.5% |
|  | GOT-ABIERO | 580 | 49 | 23 | 4.0% |
|  | RARIEDA MIX | 670 | 78 | 36 | 5.4% |
|  | RAMBA BOYS | 1140 | 125 | 33 | 2.9% |
|  | MAKASEMBO | 600 | 57 | 29 | 4.8% |
|  | NDIGWA | 550 | 69 | 39 | 7.1% |
|  |  |  |  |  |  |
|  | **COLLEGES** |  |  |  |  |
|  | BONDO TTC | 800 | 71 | 14 | 1.8% |
|  |  |  |  |  |  |
|  | **CAMP** |  |  |  |  |
|  | SAGAM MEDICAL | 120 | 45 | 10 | 8.3% |
|  |  |  | **TOTAL** | **380** |  |
