# Supplementary material for: Seroprevalence and determinants of transfusion transmissible infections among voluntary blood donors in Homabay, Kisumu and Siaya counties in western Kenya
Source: BMC Res Notes. 2018 Mar 12;11:171. doi: 10.1186/s13104-018-3276-y (PMC5848540; doi:10.1186/s13104-018-3276-y)
Supplement: Supplementary file 2 — Additional file 2. Pre-donation questionnaire. [file 13104_2018_3276_MOESM2_ESM.pdf]

Appendix VII (a): Blood donor Questionnaire

KENYA NATIONAL BLOOD TRANSFUSION SERVICE

Donation Number

DONOR QUESTIONAIRE

Clinic Venue ---------------- **County** ---------------- **Clinic Code:** ----- **Donor Number** ---------

SECTION 1: DAILY BLOOD DONOR REGISTRATION& SCREENING FORM (Donors please complete this section below)

Surname: ________________ **Other Names:** ____________ ________ **GENDER: F / M**

Student Number/ National ID Number: ______________ **Date of Birth:** -----/-----/----- **(dd/mm/yy)**

Marital Status: ***(Mark in appropriate box)***

Single	Married	Divorced/Separated	Widowed

Contact Details: Postal Address (where you would like to receive your correspondence)

______________________________ Code ______________________________

Home phone number: ------------------------ **Cell phone number:** ------------------------

Email: ------------------------------------ Residence (county) ------------------------

Level of education: None/ Primary/ Secondary/ Tertiary Occupation: ....

When did you last donate Blood? .... **Blood Group:** ....

SECTION 2: HEALTH QUESTIONNAIRE **Circle the appropriate answer**

1. Are you feeling well and in good health today?	Yes/No
2. Have you eaten in the last 6 hours?	Yes/No
3. Have you ever fainted?	Yes/No
**In the past 6 months have you:**	
4. Been ill, received any treatment or any medication?	Yes/No
5. Had any injections or vaccinations (immunizations)?	Yes/No
6. Female Donors: Have you been pregnant or breast feeding?	Yes/No
**In the past 12 months have you:**	
7. Received a blood transfusion or any blood products?	Yes/No
**Do you have or have you ever had:**	
8. Any problems with your heart or lungs e.g. asthma?	Yes/No
9. A bleeding condition or a blood disease?	Yes/No
10. Any type of cancer?	Yes/No
11. Diabetes, epilepsy or TB?	Yes/No
12. Any other long term illness<br>Please Specify	Yes/No

Appendix VII(b): Blood Donor Questionnaire

KENYA NATIONAL BLOOD TRANSFUSION SERVICE

SECTION 3: RISK ASSESSMENT QUESTIONNAIRE

The lives of patients who receive your blood are totally dependent on your honesty & frankness in answering the questions below. Your answers will be treated in a confidential manner. Circle the appropriate answer.

**In the past 12 months have you:**	
1. Received or given money, goods or favours in exchange for sexual activities?	Yes/No
2. Had sexual activity with a person whose background you do not know?	Yes/No
3. Been raped or sodomized?	Yes/No
4. Had a stab wound or had an accidental needle stick injury e.g. injection needle?	Yes/No
5. Had any tattooing or body piercing e.g. ear piercing?	Yes/No
6. Had a sexually transmitted disease (STD)?	Yes/No
7. Live with or had sexual contact with someone with yellow eyes or yellow skin?	Yes/No
8. Had sexual activity with anyone besides your regular sex partner?	Yes/No
**Have you ever:**	
9. Had yellow eyes or yellow skin?	Yes/No
10. Injected yourself or been injected, besides in a health facility?	Yes/No
11. Used non-medical drugs such as Marijuana, Cocaine etc?	Yes/No
12. Have you or your partner been tested for HIV?	Yes/No
13. **Do you consider your blood safe to transfuse to a patient?**	Yes/No

SECTION 4: DECLARATION (Please read this before you complete the form with your name and signature below)

I declare that I have answered all the questions truthfully and accurately.

I understand that my blood will be tested for HIV, Hepatitis B & C, and Syphilis and the results of my tests may be obtained from the National Blood Transfusion Service.

I understand that should any of the screening tests give a reactive result, I will be contacted by use any communication medium(s) to send me **important information**. Such medium(s) shall include but not limited to e-mail, post office, mobile telephone and/or fixed telephone, and offered counseling to make an informed decision about further confirmatory testing and management.

I hereby give consent to KNBTS to use the contact details provided in this form to communicate to me as the need may be.

I understand the blood may be used for scientific research, main objective being to improve the safety of the blood supply to patients.

I consent to give blood; I understand that it may be used for transfusion for the benefit of others.

Signature: -- **Date:** ----------------------------------

For Official Use:

Weight (kg)	Hb >12.5g/dl	BP	Pulse

Donor is Accepted	
Yes	No

Report:

Name of Nurse / Counselor: ---------------------------------- **Date:** ----------------------------------

Low Volume	> 1 Venepuncture	Hematoma	Faint		
			Mild	Moderate	Severe

Time Needle In		Time Needle Out	

**Report:**<br>**Name of Phelotomist**	**Date**

Page 2 of 2 **FRM CLN 001** **Revision 02**
